# The Metaphysical Magazine

**Published:** 1896-07

**Authors:** 


THE METAPHYSICAL MAGAZINE, published by the Metaphysical Publishing Company, 503 Fifth Avenue, New York, for July, is out. Price, 25 cents; yearly subscriptions, $2.50; in foreign countries, 14*s*.

Among other articles Professor Elmer Gates, formerly of the Smithsonian Institution, explains for the first time the results of his extended experimental researches in the domain of Psychology. These experiments have been conducted in a thoroughly scientific manner, and the demonstrations are of the very highest importance to every branch of learning.

The contents of this number also include: "Karma in the Bhagavad Gita," by Charles Johnston, M. R. A. S.; "The Subtile Body," by E. G. Day, M. D.; "The Serpent and Its Symbol," by Lieut. C. A. Foster, U. S. N.; "Spirit in Man and Nature," by C. Staniland Wake; "Conception and Realization of Truth," by Frank H. Sprague; "A Prophetess of the New Life," by Lilian Whiting; and other articles on occult, philosophic, and scientific lines.

